# Supplementary material for: Reconfigurable infrared hyperbolic metasurfaces using phase change materials
Source: Nat Commun. 2018 Oct 22;9:4371. doi: 10.1038/s41467-018-06858-y (PMC6197242; doi:10.1038/s41467-018-06858-y)
Supplement: Supplementary file 1 — Supplementary Information [file 41467_2018_6858_MOESM1_ESM.docx]

Supplementary Information:

Reconfigurable Mid-Infrared Hyperbolic Metasurfaces using Phase Change Materials

## T. G. Folland et. al.

## Supplementary Note 1: Launching and Reflecting Hyperbolic Polaritons at Interfaces

Our sample presents three different interfaces, each of which can have distinctive properties in terms of launching polaritons in the s-SNOM experiment. Observing a tip-launched mode requires a strong reflection from an interface, while observation of an edge-launched mode demands strong scattering off the sample edge. First, we consider the edge of the hBN flake. The polariton cannot propagate past the edge of the flake and therefore nearly 100% is reflected, leading to a strong tip-launched mode. On the other hand, these hBN flakes are thin (24 nm), and therefore interact only weakly with incident waves, suppressing the edge-launched mode (similar to Ref 25 main text).^1^ Thus, we only observe the tip-launched mode near the hBN crystal edge.

Second, there is an interface where the hBN extends over the edge of the VO_2_ crystal. As the films of hBN are continuous across the VO_2_ edge, tip-launched modes can propagate over this interface and will only be weakly reflected. This has been observed in earlier experiments, for example Ref. 25 of the main text.^1^ On the other hand, the VO_2_ crystal itself strongly scatters incident waves to launch polaritons from the VO_2_ crystal edges. Therefore, we only see the edge-launched modes at the interfaces between hBN and the VO_2_ crystal.

The third type of interface is the domain boundaries between dielectric and metallic VO_2_. Due to the relatively small size of the domains in this sample, these show much weaker s-SNOM signals, however, the same arguments as for the edge of the VO_2_ crystal hold. Therefore, we mainly see the edge-launched polaritons. This hypothesis is qualitatively supported by electromagnetic simulations of plane waves incident on these three types of boundaries, presented in Supplementary Fig. 1. The results show that polaritons launched from the VO_2_ crystal edge (Supplementary Fig. 1a) or dielectric-metal domain boundaries (Supplementary Fig. 1b) are relatively strong, whilst those initiated from the edge of the hBN flake (Supplementary Fig. 1c) are relatively weak in intensity.

For the experimental efforts exploring the changes in polariton wavelength, refraction and propagation over the two VO_2_ domain types, we employed a series of heating and cooling cycles with s-SNOM measurements performed at various temperatures and incident frequencies. The generalized process for these measurements is summarized in Fig. S2, whereby the sample was initial measured using S-SNOM at room temperature, then heated to various temperatures just below, within and then above the phase-change temperature, with s-SNOM measurements performed at specific temperatures within this range. Following these efforts, the sample was cooled, thereby resetting the VO_2_ to the dielectric phase, where the process could be repeated with different phase change domain structures. Multiple heating and cooling cycles were performed for the experiments discussed in this work, with no changes in the response of the dielectric functions of the constituent materials observed, thereby illustrating the reproducibility of this process.


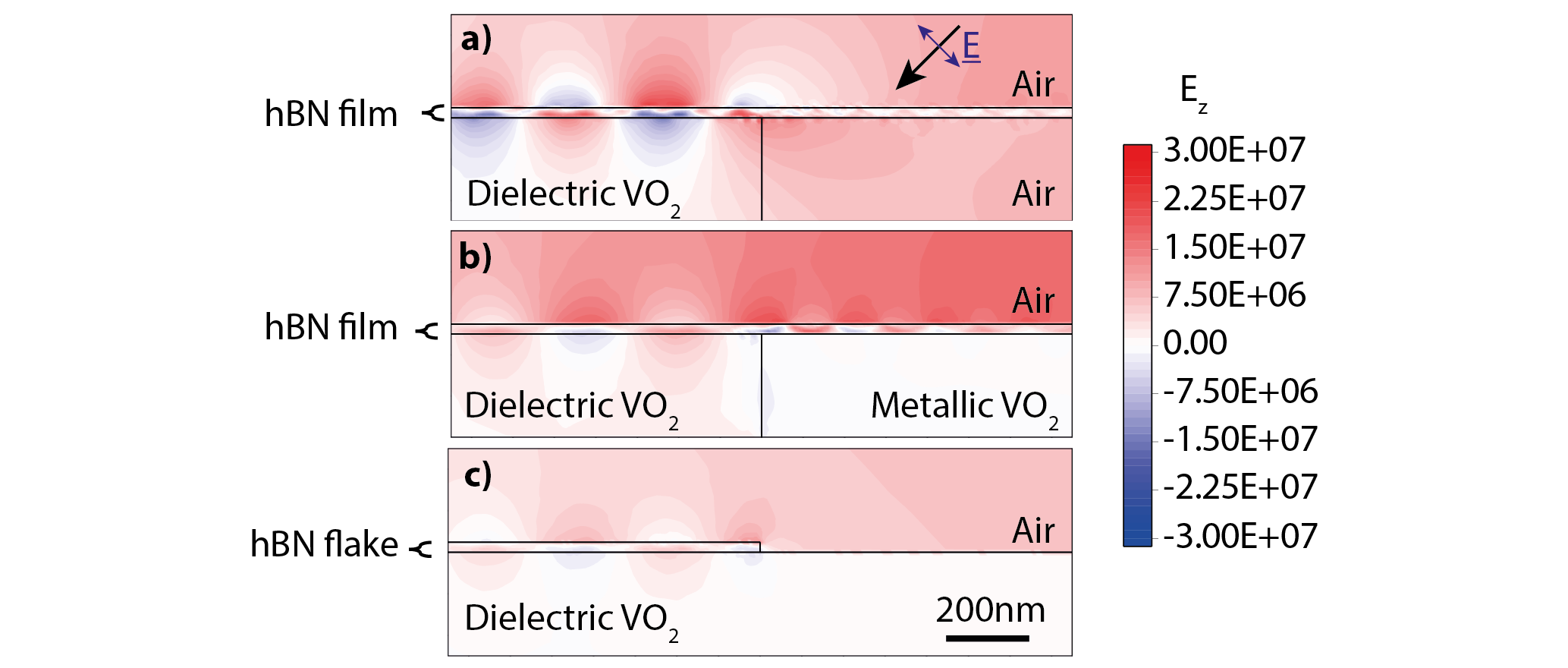


Supplementary Figure 1: Cross sectional plot of electromagnetic fields from hyperbolic polaritons launched at the interface between a) dielectric VO_2_ and vacuum, b) dielectric VO_2_ and metallic VO_2_ and c) at the edge of a hBN flake on VO_2_. The peak electromagnetic fields launched above the dielectric domain are ~2.3·10^7^ V/m, ~1.8·10^7^ V/m and ~1.2·10^7^ V/m, indicating the strongest fields are launched at the edge of the VO_2_ flake, however, these simulations clearly show that edge-launched modes are highly suppressed in c).

##
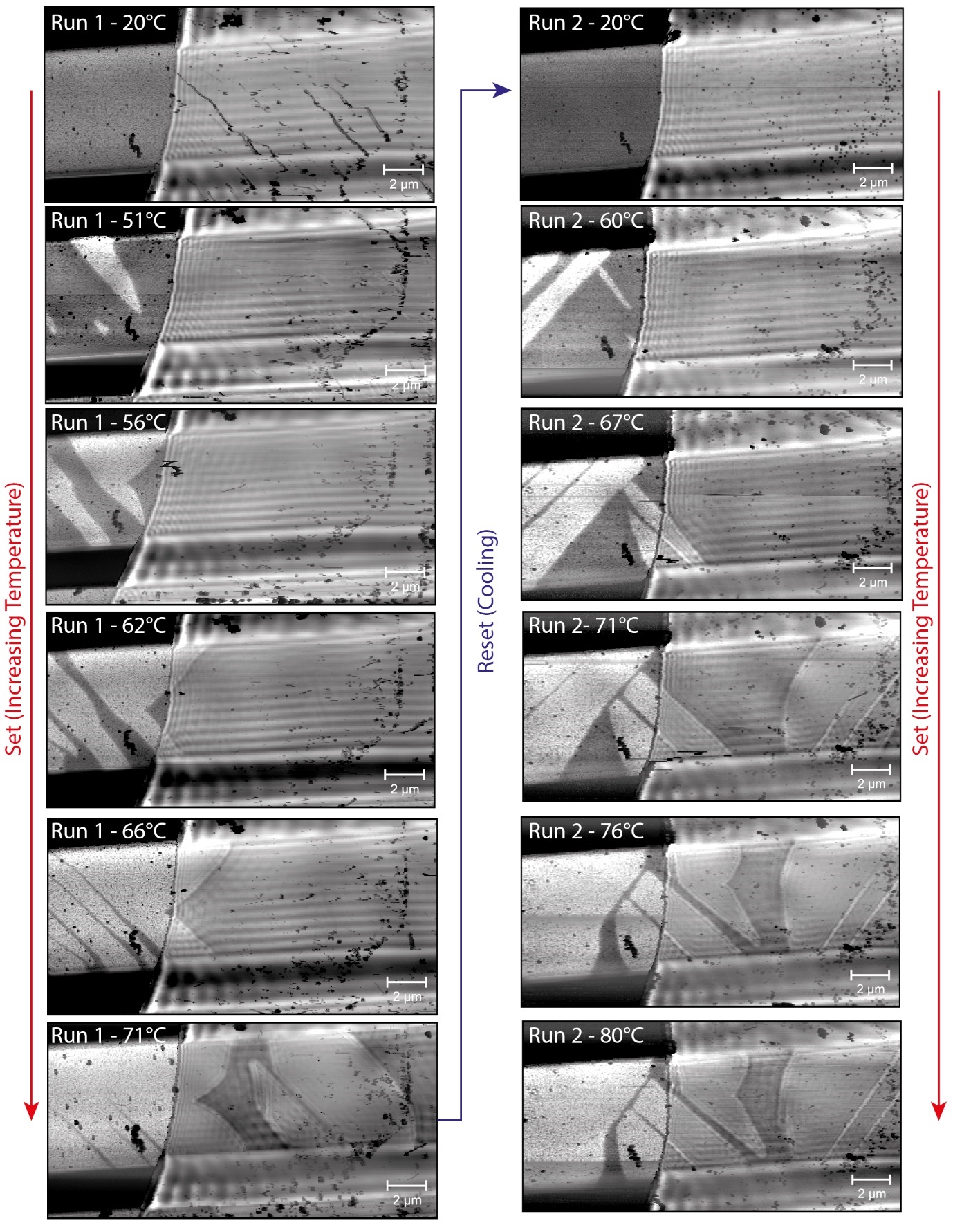


Supplementary Figure 2: Thermally induced phase transition in VO_2_ for reconfigurable metasurfaces. Here we show a series of s-SNOM images taken at the same position as the sample temperature is increased, showing the growth of metallic VO_2_ domains, which manipulate polariton propagation in hBN. By cooling the device back to room temperature the device is reset to its dielectric state, and, upon reheating, form a different phase domain pattern.

## Supplementary Note 2: Absence of Out-of-Plane Topographic Change during Crystal VO_2_ Phase Transition


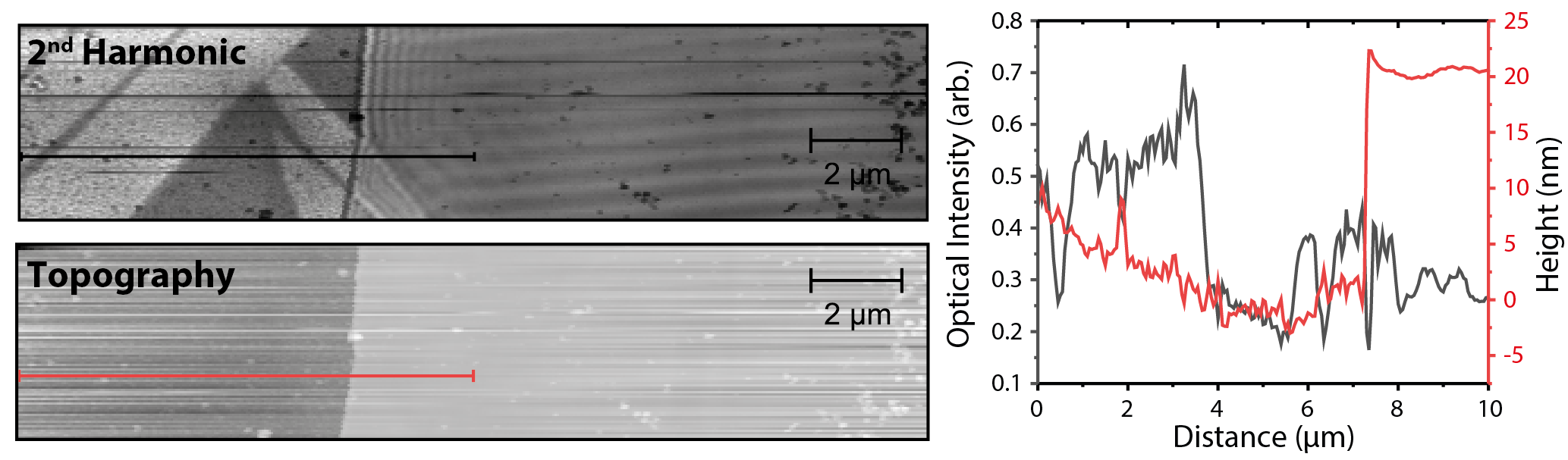


Supplementary Figure 3: Topographic variation upon phase transition. Near-field 2^nd^ Harmonic IR amplitude (left top) and topography (left bottom), with the correlated line profiles provided on the right (red - IR amplitude and black - topography).

To show that the observed optical effects are attributable to changes in the dielectric environment and not to topographical changes along the out-of-plane axis, we compared spatial maps of the sample with optical s-SNOM measurements (Supplementary Fig. 3). Comparing line scans at the same topographical location in both 2^nd^ harmonic IR amplitude s-SNOM signal and topographical height, we see that while there appears to be small topographic variations (due to noise induced at elevated temperatures, and residual contaminants from the device fabrication process), these do not correlate with the location of domains in the IR s-SNOM amplitude maps. This clearly demonstrates that the domains observed in the IR s-SNOM maps are due to the change in the phase change material (PCM) dielectric function, not to changes in the topography of the sample surface.

## Supplementary Note 3: Additional images of polariton refraction and determination of dispersion

Experimental demonstration of HPhP refraction for polaritons transmitted across a dielectric-metallic domain was presented in Fig. 2a,b of the main text. For completeness and to demonstrate the how the additional refraction angles that were reported in Fig. 3c, we provide three additional s-SNOM maps that were collected at the same incident frequency as Fig. 2a,b, but were collected during different thermal cycles, providing different domain angles with respect to the VO_2_ crystal edge. In all cases, the HPhP launching within the metallic domain from the VO_2_ crystal edge is designated with the red lines, while the refracted HPhP by the black lines. The corresponding linescans extracted from these images are provided below each s-SNOM map and demonstrate again that despite the refracted wave propagated at a direction that is non-normal to either the VO_2_ crystal or PCM domain edge that it has the same HPhP wavelength (black curves) as the edge-launched mode in the same domain (red curves).


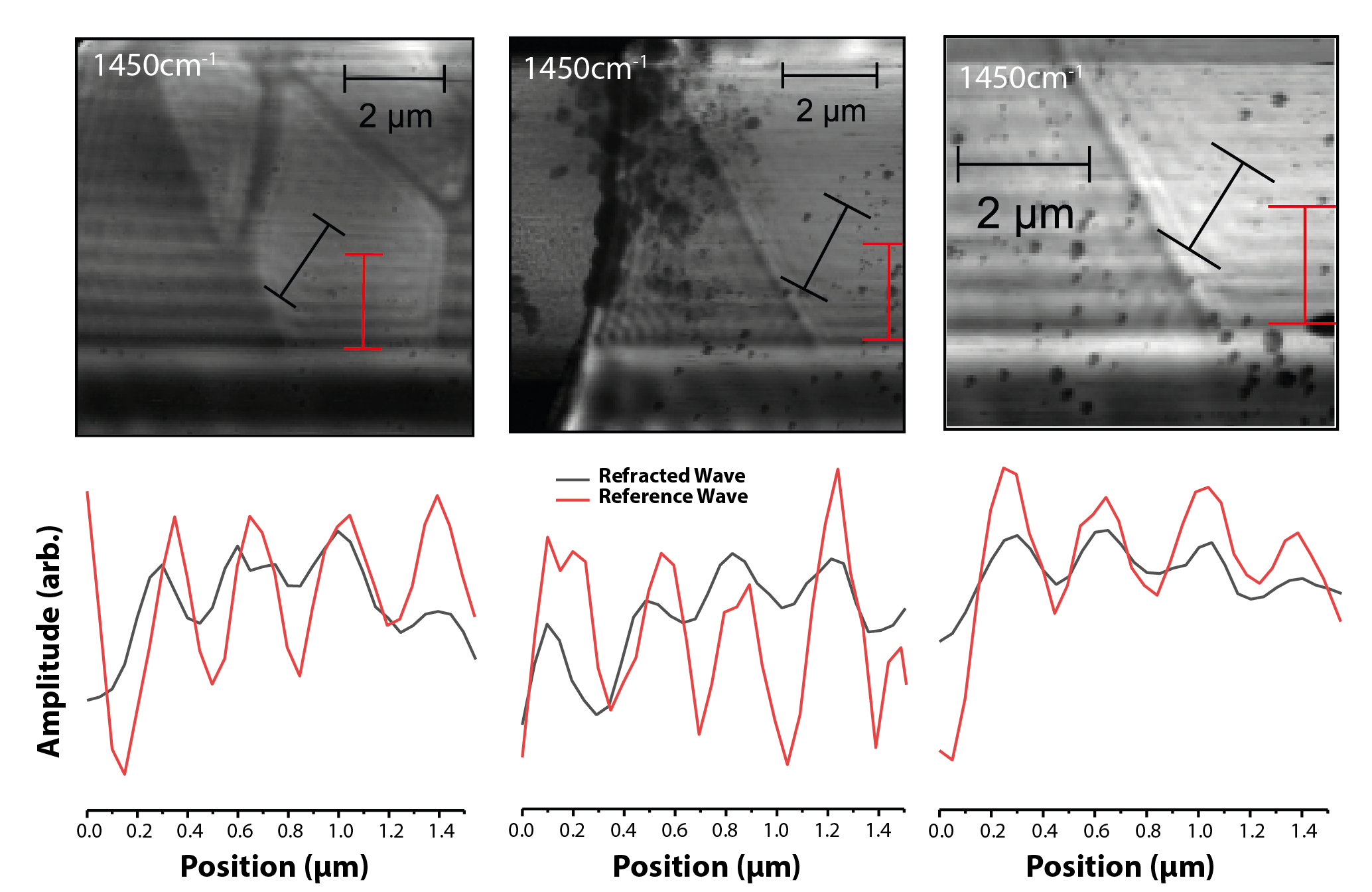


**a)**

Supplementary Figure 4: Additional images of polariton refraction in S-SNOM experiments. Dark patches on the images are contaminants that built up on the sample due to continuous imaging at high temperatures. The s-SNOM maps were collected at 1450cm^-1^ frequency and 65.5,58 and 55.7°C temperatures.

The extraction of the HPhP wavelength was performed as described within the main text and methods sections. This is also illustrated in Supplementary Fig. 5, where we present a) the s-SNOM maps collected at two different incident frequencies (as labelled) when the underlying VO_2_ was in the dielectric (top two) and metallic (bottom two) phase. Linescans were collected along the trajectories designated by the white lines in Supplementary Fig. 5a, and are presented for each plot in Supplementary Fig. 5b. Through implementing a discrete Fourier transform (FFT) of these linescans, the frequency component of the HPhP propagation and thus, the HPhP wavelength within these domains can be extracted (Supplementary Figure 5c). By plotting the frequency dependence of this wavelength for both VO_2_ phases, the dispersion plots presented in Fig. 3a and b, respectively, were realized.


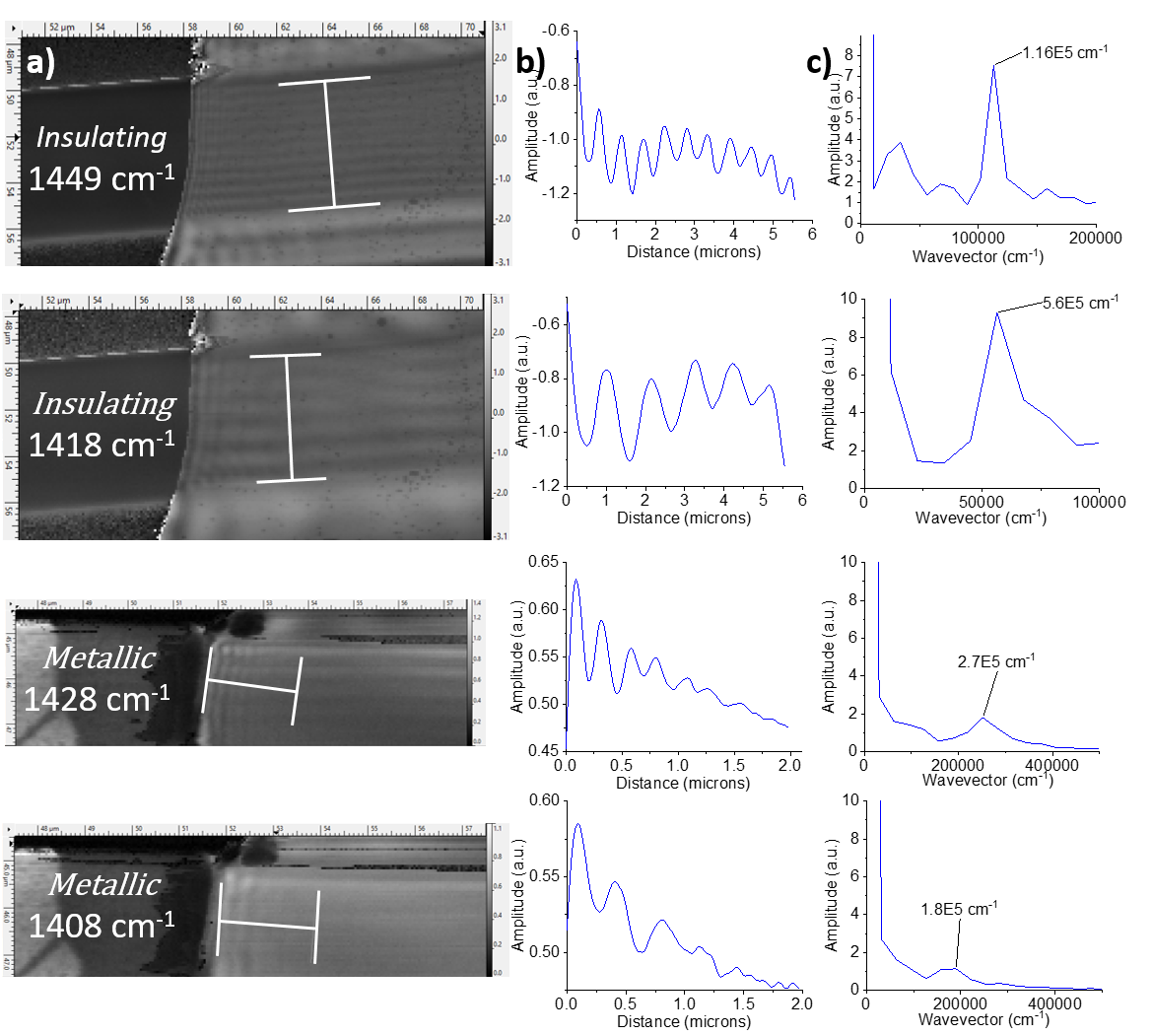


Supplementary Figure 5: Determining the polariton wavelength for given VO_2_ phases and laser frequencies. a) The s-SNOM amplitude corresponds to line-scan markers perpendicular to the dominant propagating wave. b) The line-scan profiles, taken where shown in **a**). c**)** The frequency component of each line scan was extracted using fast Fourier transforms (FFTs) with a rectangular window, and normalizing amplitude to the mean-square amplitude (MSA). The peaks show the dominant frequencies in the line scan. Peak positions were converted to wavevector and used to plot the dispersions in Fig. 3.

## Supplementary Note 5: Electromagnetic Simulations of Polariton Refraction

To supplement experimental results on polariton refraction, we conducted electromagnetic simulations for various metallic-dielectric domain angles and frequencies, identical to Fig. 2c, and present them in Supplementary Fig. 6a. In each simulation, the angle of the refracted wave was determined by inspection, from which we plotted Supplementary Fig. 6b, analogous to Fig. 3c. Lines in Supplementary Fig. 6b were extracted by calculating $n_{1}$ and $n_{2}$ for the polaritons propagating over both the metallic and dielectric domains from the polariton wavelength. As a result, the plots in Supplementary Figure 6b contains no fitting, and shows excellent agreement with Snell’s law.


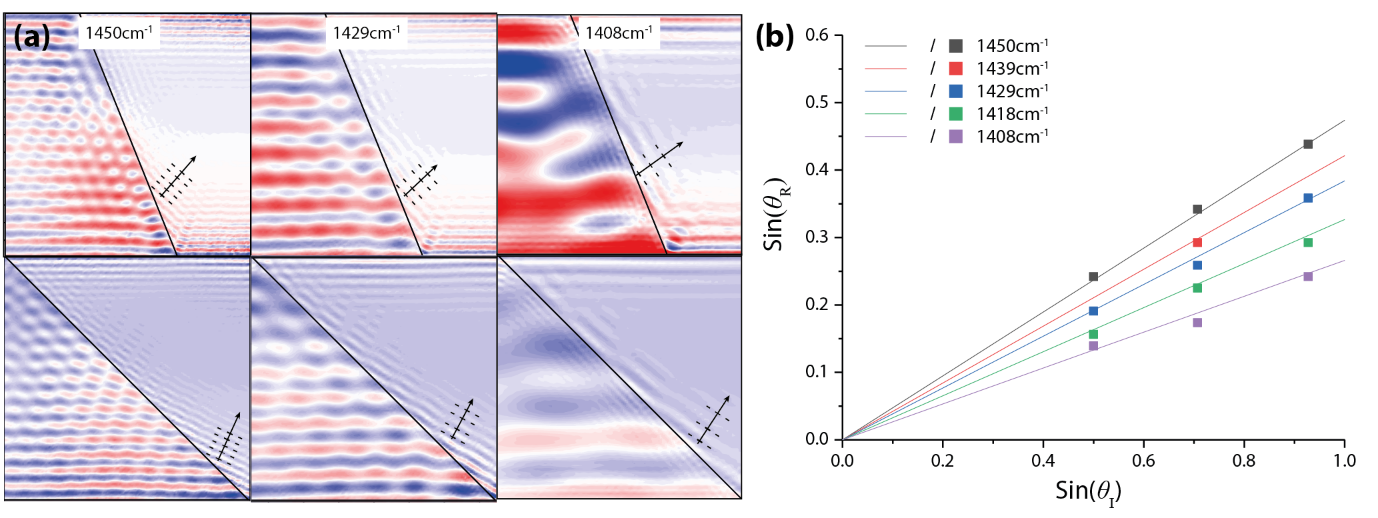


Supplementary Figure 6: a) Electromagnetic simulations of polariton refraction at 68˚ and 45˚ with respect to the surface normal. Each image shows the refracted wave, and was used to create the data points in b). b) Snell’s law tested using electromagnetic simulations. The calculations follow Snell’s law closely, with discrepancies due to uncertainties in accurately determining refraction angles.

## Supplementary Note 6: VO_2_ and hBN rewritable metasurface

In Fig. 4a of the main text, we present the simulated reflectance spectrum of a hBN film on top of VO_2_ patterned into metallic and dielectric domains (hereafter referred to as VO_2_ resonators). Here we address the frequency tuning of the resonances observed in these spectra. We address two approaches to achieving frequency tuning – changing resonator size $L$ ($L$=250 nm in Fig. 4a) with fixed pitch $P$ ($P$=500 nm in Fig. 4a) and changing pitch with fixed filling fraction ($f=L/P$). The reflectance spectrum of such reconfigurable resonators as a function of the resonator size, is provided in Supplementary Fig. 7a and illustrates both a variation in reflectance, and small changes in the spectral positions of the resonant modes. The change in the overall reflectance can be attributed largely to changes in the reflectance of the VO_2_ resonators, with larger resonators exhibiting higher reflection. The spectral mode shifts are approximately 3.34, 3.34, 4 and 2 cm^-1^ from lowest to highest modal wavenumbers, which is much lower than what would be expected for localized resonances. The reason for this becomes clear when we consider the influence of grating pitch with a constant fill fraction in Supplementary Fig. 7b. Here we see that each mode red-shifts significantly (approximately 30 cm^-1^ for the mode around 1525 cm^-1^) with increasing grating pitch. Note that here the overall reflectance does not change significantly, as the fraction of metallic vs dielectric VO_2_ remains approximately constant when we fix the fill fraction. Our resonant tuning behaviour is consistent with our metasurface effectively acting as a grating coupler, as HPhPs can freely propagate through the hBN film. We note that while here we do not show significant absorption or reflection resonances induced by the metasurface design, this could potentially be achieved by optimizing the combination of hBN thickness, VO_2_ crystal thickness and lateral size, along with the designed metallic domain pitch and size.


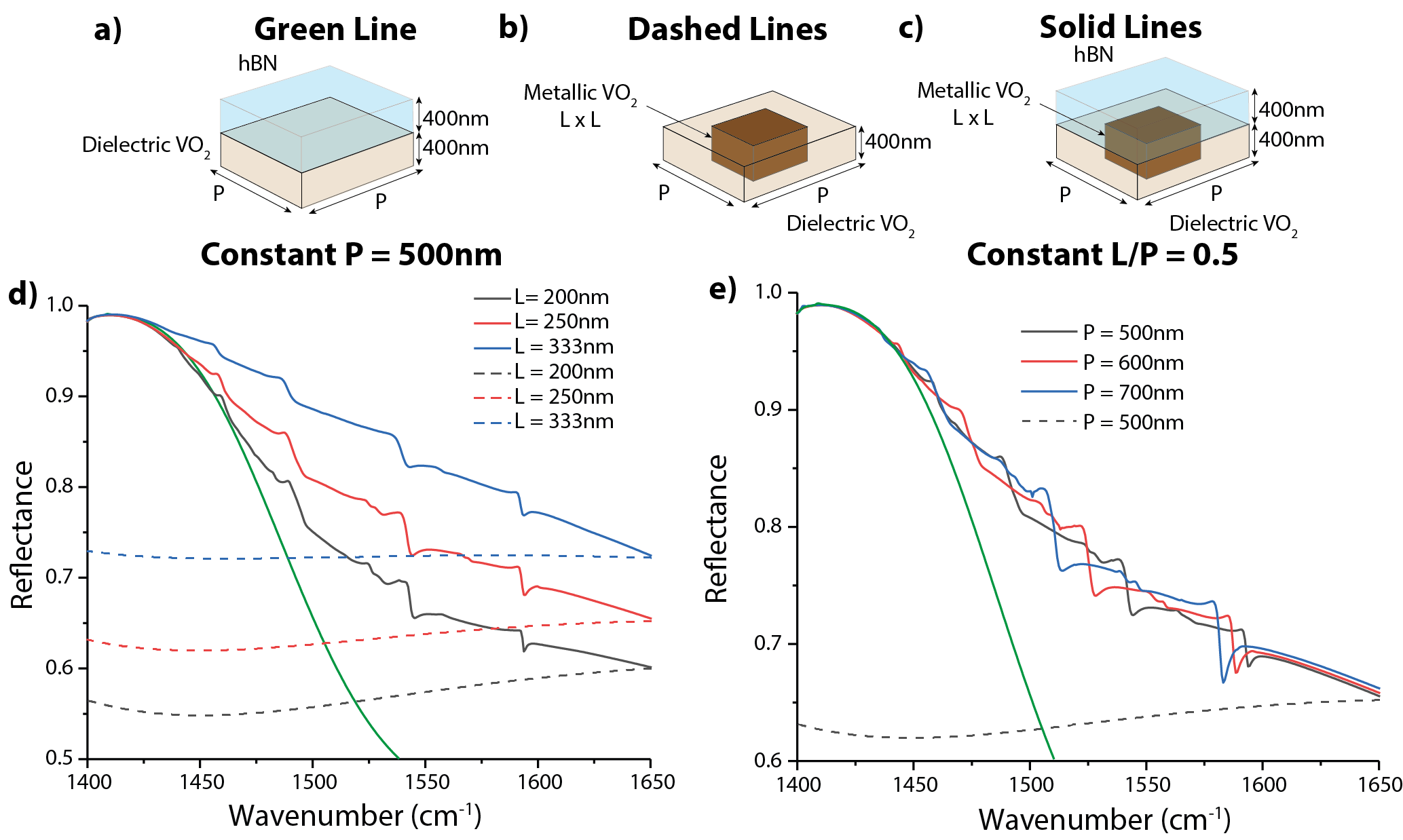


Supplementary Figure 7: Frequency tuning of hyperbolic modes for the metasurface presented in Fig 4a. a) Shows tuning of the resonant modes for different metallic domain sizes at constant pitch, b) the tuning of the resonant modes for variable domain pitch.

## Supplementary Note 7: VO_2_ and GeSbTe hyperbolic waveguides


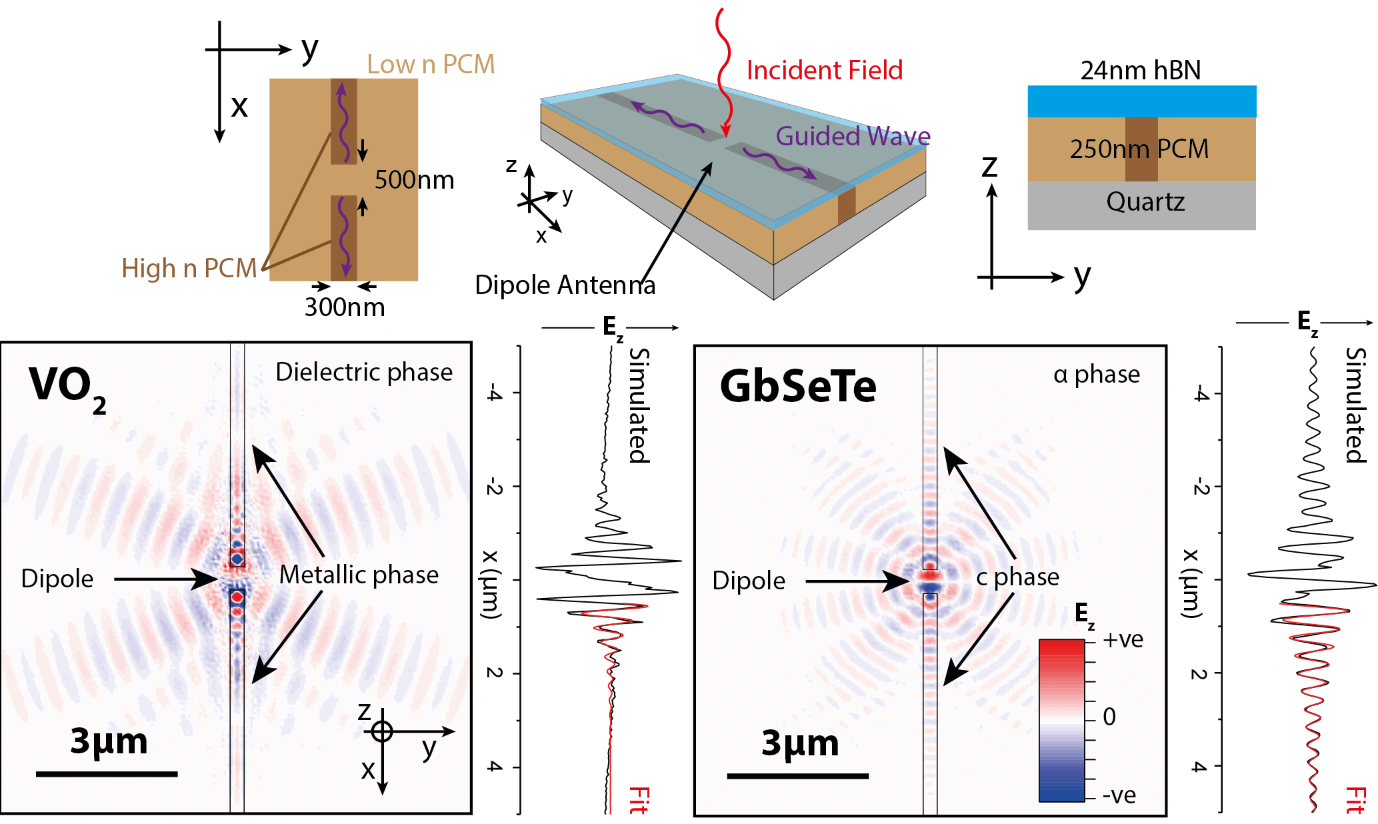


Supplementary Figure 8: Simulation of a near-field waveguide achieved using phase-change materials. Bottom shows E_z_ electric field profiles taken from the centre of the hBN, with extracted line profiles from the centre of the waveguide. Red lines show fitting of a damped sine wave to the simulated data.

Here we compare the performance of VO_2_ and GeSbTe PCMs for applications in creating hyperbolic polariton waveguides. Whilst the phases of VO_2_ are discussed in the main text, GeSbTe also possesses both metallic and dielectric phases, which can be cycled by heating and cooling. In contrast to VO_2_, the dielectric phase of GeSbTe is amorphous (α-phase), and the metallic phase is cubic (c-phase). To simulate a hyperbolic waveguide in both materials, a 300 nm metallic/c-phase domain was formed within a dielectric/α-phase of VO_2_/GeSbTe (see Supplementary Fig. 8). This forms a lateral waveguide, where the high refractive index of the polariton within the strip prevents light from escaping. A small gap in the metallic phase (500 nm wide) creates an antenna that couples far-field waves into both the laterally confined waveguide mode and a radially propagating mode. For the VO_2_ structure the radial wave propagates a significant distance over the dielectric domain, due to low losses inherent to the dielectric medium. However, in metallic VO_2_ the guided wave is suppressed after just a few oscillations, with a $1/e$ propagation length of $\gamma=$ 0.57 µm extracted by fitting a decaying sine wave. In contrast, for GeSbTe the waveguide mode appears to propagate a longer distance than the radial mode, with a decay length of $\gamma=$1.57µm. This shows that the phenomena reported in this paper should be observable in GeSbTe films, and that this PCM might be better suited to some waveguide applications, In principle, by optimizing the waveguide width, hBN and PCM thickness it may be possible to optimize this structure to achieve long range (>$\lambda$) propagation of the polariton mode.

**Supplementary References**

1 Duan, J. *et al.* Launching phonon polaritons by natural boron nitride wrinkles with modifiable dispersion by dielectric environments. *Adv. Mater.* **29**, 1702494 (2017).
